# Reconsidering the diagnostic potential of thyroperoxidase autoantibodies in chronic spontaneous urticaria^☆^

**DOI:** 10.1016/j.waojou.2025.101144

**Published:** 2025-11-26

**Authors:** Parth Aphale, Himanshu Shekhar, Shashank Dokania

**Affiliations:** Dr. D.Y. Patil Vidyapeeth (Deemed to be University), Pimpri, Pune, Maharashtra, India

Dear Editor,

We read with great interest the article by López et al on the “Association between circulating thyroperoxidase (TPO), anti-TPO IgE, anti-TPO IgG, and different clinical outcomes in chronic urticaria” (World Allergy Organization Journal, 2025).^1^ The authors present a hypothesis-generating study exploring the interplay between TPO and its autoantibodies in chronic spontaneous urticaria (CSU), an area of growing attention given the complex overlap between type I autoallergy and type IIb autoimmunity. While the findings are thought-provoking, we would like to offer some constructive critique and raise questions for future exploration.

First, the study highlights that circulating TPO levels did not correlate with the presence of autoantibodies or clinical outcomes, whereas distinct clinical clusters emerged with anti-TPO IgE and IgG positivity. This raises the issue of temporal dynamics in biomarker expression. Autoantibody levels may fluctuate with disease activity and treatment exposure, and cross-sectional measurement may underestimate their diagnostic or prognostic value. Would serial sampling across disease flares versus remission better capture the relevance of these markers?^2^

Second, the use of a home-based ELISA for anti-TPO IgE, although previously validated by the authors, poses questions regarding reproducibility across centers. Lack of standardized assays remains a key barrier to the clinical implementation of autoallergic biomarkers. A multicenter effort toward harmonization of laboratory protocols, akin to initiatives in autoimmune thyroid disease testing, may accelerate translation of these findings.^3^

Third, while the study reports minimal overlap between anti-TPO IgE and IgG, the possibility of shared epitopes cannot be dismissed. Prior reports suggest that co-occurrence of IgE and IgG autoantibodies, although infrequent, may denote a subgroup with more severe or treatment-resistant CSU. Could these rare patients represent a transitional phenotype bridging type I and type IIb autoimmunity?^4^

Furthermore, clinical applicability should be carefully weighed. Anti-TPO IgG is already recognized by international urticaria guidelines as supportive of type IIb autoimmunity, yet its sensitivity and specificity remain suboptimal. The authors’ finding that anti-TPO IgE associates with angioedema and NSAID hypersensitivity is intriguing and clinically relevant, but replication in larger, multi-ethnic cohorts is essential to ensure external validity.

Finally, the study suggests that TPO itself does not directly drive mast cell activation, aligning with mechanistic evidence from Mozena et al. (2010). This underlines that autoantibodies, rather than the antigen, may be the functional drivers of pathogenesis. Could therapeutic strategies targeting autoantibody production (eg, B cell depletion or IgE neutralization) be more promising than antigen-based interventions in this subgroup of CSU?^5^

In conclusion, López et al provide an important foundation for disentangling the heterogeneity of CSU endotypes. To enhance clinical translatability, future studies should emphasize longitudinal biomarker assessment, inter-laboratory assay standardization, and phenotype-specific therapeutic response evaluation.

## Funding and disclosures

None.

## CRediT authorship contribution statement

**HS**- Conception and Design, Final review, Conceptualization. **PA**- Data acquisition, Writing the manuscript. **SD**- Analysis, Interpretation.

## Declaration of generative AI

The authors declare that AI-based language assistance was used solely for grammar refinement.

All intellectual content, interpretation, and conclusions are the sole responsibility of the authors.
